# Current understanding of the bi-directional relationship of major depression with inflammation

**DOI:** 10.1186/2045-5380-2-4

**Published:** 2012-02-28

**Authors:** Berhane Messay, Alvin Lim, Anna L Marsland

**Affiliations:** 1 Department of Psychology, University of Pittsburgh, 3213 Sennott Square, 210 S. Bouquet St., Pittsburgh, PA 15260, USA

**Keywords:** depression, negative affect, inflammation, inflammatory markers, cytokines

## Abstract

Consistent evidence links major depression and its affective components to negative health outcomes. Although the pathways of these effects are likely complex and multifactorial, recent evidence suggests that innate inflammatory processes may play a role. An overview of current literature suggests that pathways between negative moods and inflammation are bi-directional. Indeed, negative moods activate peripheral physiologic mechanisms that result in an up regulation of systemic levels of inflammation. Conversely, peripheral inflammatory mediators signal the brain to affect behavioral, affective and cognitive changes that are consistent with symptoms of major depressive disorder. It is likely that these pathways are part of a complex feedback loop that involves the nervous, endocrine, and immune systems and plays a role in the modulation of peripheral inflammatory responses to central and peripheral stimuli, in central responses to peripheral immune activation and in the maintenance of homeostatic balance. Further research is warranted to fully understand the role of central processes in this feedback loop, which likely contributes to the pathophysiology of mental and physical health.

## Background

Evidence shows an association of major depression with increased risk for adverse physical health outcomes. Indeed, depression, whether assessed as a continuum of symptoms or as the presence of a clinical syndrome, predicts the incidence and progression of diseases of aging, including cardiovascular, metabolic and neurodegenerative diseases, as well as all-cause mortality [1-3]. Given the burden of these physical illnesses, it is not surprising that affective symptoms and disorders are more prevalent among the medically-ill than the general population [4,5], raising the possibility that associations between affective disorders and physical health are bi-directional in nature. It is also likely that lifestyle choices contribute to poorer health among individuals with depression [6]; however, to date, evidence suggests that behavioral factors contribute only minimally to depression-related variability in health risk. Thus, other mechanisms must also be operating. Accumulating evidence suggests that the immune system may play a role.

### Main Text

Early studies show an association of depression with the down-regulation of functional parameters of the immune system (for example, decreased ability of NK cells to destroy tumor cells [7]). However, this immune suppression is not as 'global' as initially proposed. Indeed, recent attention has focused on the activation of innate, non-specific inflammatory mechanisms that also accompany depressed mood [8]. These differential immune responses to negative mood have been interpreted within an evolutionary context as a down-regulation of processes that take time and energy in favor of an up-regulation of processes that are immediately available to defend the organism [9]. Although adaptive and of potential health benefit in the short-term, growing evidence shows that chronic elevation of inflammation plays a role in the pathogenesis and course of numerous age-related physical health conditions, possibly contributing to the co-morbidity of depression with chronic physical illness [10].

The inflammatory response is a non-specific immune reaction that is initiated when monocytes/macrophages are activated by pathogens or tissue damage to release pro-inflammatory cytokines, such as interleukin (IL)-6, IL-1β, and tumor necrosis factor (TNF)-α. These cytokines initiate a local and systemic inflammatory response, which includes the hepatic synthesis and release of acute phase proteins, such as C-reactive protein (CRP) and fibrinogen [11]. Peripheral pro-inflammatory cytokines also signal the brain, resulting in symptoms of sickness that typically accompany infectious disease, such as fever, depressed affect, suppressed appetite, increased sleep, and cognitive deficits [12,13].

Circulating levels of pro-inflammatory mediators are widely accepted as a marker of systemic levels of inflammation. However, caution should be taken in assuming these inflammatory mediators are immune-derived as many cells produce these signaling proteins, including adipocytes and endothelial cells [14]. Regardless of source, circulating levels of IL-6 are relatively stable over extended periods [15], are positively related to age [16], and predict risk for a range of age-associated diseases [17]. Consistent evidence also shows that individuals with major depressive disorder have higher levels of circulating markers of inflammation than non-depressed individuals. For example, two recent meta-analyses concluded that increased plasma levels of TNF-α, IL-6, IL-1, and CRP accompany major depression [18,19].

Available data suggest that relationships between pro-inflammatory cytokines and depressed mood are bi-directional. In support of immune-to-brain pathways, sickness symptoms mediated by increases in circulating pro-inflammatory cytokines are consistent with symptoms of depression including fatigue, sleep disturbances, anxiety, negative mood, anhedonia, and loss of appetite [20]. Indeed, the experimental or clinical administration of cytokines or endotoxins results in a range of symptoms of depression [21]. For example, clinical administration of the pro-inflammatory cytokine interferon (IFN)-α in the treatment of cancer or chronic infection induces symptoms of major depressive disorder in 23% to 45% of all patients, with the degree of depression being positively related to dose and duration of treatment [22]. Epidemiologic evidence also shows that systemic inflammation predicts future risk for depressive symptoms and clinical episodes of depression in some [23-25], but not all longitudinal studies [26,27].

To examine the impact of peripheral inflammation on the central nervous system, recent attention has focused on whether immune-related patterns of brain activation are consistent with those that accompany clinical depression. Here, animal studies show that pro-inflammatory cytokines can penetrate the blood-brain barrier to stimulate the production of central pro-inflammatory cytokines by microglial cells in discrete brain regions that are involved in mood regulation and reward processing [20]. Recent human studies have employed randomized double-blind trials, exposing subjects to either immune stimulants (usually endotoxin) that generate low-grade systemic inflammatory responses or saline placebo and then comparing patterns of brain activation across the groups using functional magnetic resonance imagery. Using these methods, peripheral inflammation has been associated with negative mood states that are accompanied by increased activation of the subgenual anterior cingulate cortex (sgACC) and decreased connectivity of the sgACC with the amygdala, prefrontal cortex, nucleus accumbens, and superior temporal sulcus in response to emotional stimuli [28]. A similar pattern of heightened sgACC activity has been observed in response to IFN-α treatment [29] and during episodes of major depression, with activity returning to normative levels once symptoms remit [30]. Peripheral administration of endotoxin also reduces activity in the ventral striatum in response to a monetary reward task, a region of the brain implicated in the pleasurable effects of reward [31]. Taken together, these results raise the possibility that inflammation plays a role in the pathophysiology of the affective and anhedonic symptoms of depression [32].

In addition to immune-to-brain pathways, evidence also shows that negative mood states, stressful experiences, and antagonistic dispositions can activate peripheral physiologic pathways that modulate immune function. For example, negative moods are associated with activation of the hypothalamic-pituitary-adrenal (HPA) axis and the peripheral release of cortisol, along with increased activation of the sympathetic and decreased activation of the parasympathetic branches of the autonomic nervous system [33,34]. These physiologic responses modulate the activity of immune cells and are associated with increased production of pro-inflammatory cytokines [35] and levels of systemic inflammation [36]. Indeed, symptoms of depression have been positively associated with production of TNF-α by monocytes of healthy men and women [37,38]. Furthermore, some longitudinal studies show that symptoms of depression precede increases in systemic inflammation rather than result from them [26,39,27].

## Conclusion

In sum, converging evidence supports reciprocal pathways linking inflammation and the disruption of mood. It is likely that these pathways are part of a complex feedback loop that involves the neuroendocrine and immune systems and plays a role in both the modulation of peripheral inflammatory responses to stimuli and the maintenance of homeostatic balance. Further research examining specific symptoms and the central processes involved in these circuits is warranted to fully understand the role of inflammation in the pathophysiology of depression and of associated health morbidity. Evidence suggests that examining the impact of peripheral inflammation on central processes that play a role in specific symptoms of depression may point to novel targets for future intervention. For instance, Motivala *et al. *[40] found that changes in sleep during depression shared a stronger association with peripheral pro-inflammatory cytokines than other symptoms. Further research is also warranted to examine whether age-related increases in systemic inflammation impact brain function and thus contribute to late-life depression.

## Competing interests

The authors declare that they have no competing interests.

## Authors' contributions

BM conducted an up to date review of the relevant literature and wrote the initial draft of the manuscript. ALM edited the manuscript and made substantial revisions. AL read, contributed relevant research, and provided feedback on early and final versions of the manuscript. All authors read and approved the final manuscript.

## Authors' information

ALM: Anna Marsland, Ph.D. is the Director of the Behavioral Immunology Laboratory and a member of the Biological and Health Psychology faculty at the University of Pittsburgh. Her expertise is in the field of psychoneuroimmunology. BM: Berhane Messay is a second year graduate student in the Clinical and Biological/Health Psychology Programs at the University of Pittsburgh. AL: Alvin Lim is a second year graduate student in the Biological/Health Psychology Program at the University of Pittsburgh.
